# EFFECT OF FREQUENCY OF PARTICIPATING IN THE REGION ACTIVITY ON FUNCTIONAL DECLINE

**DOI:** 10.1093/geroni/igz038.454

**Published:** 2019-11-08

**Authors:** Masashi Yasunaga, Hisashi Kawai, Hirohiko Hirano, Hiroyuki Suzuki, Yoshinori Fujiwara, shuichi Obuchi

**Affiliations:** Tokyo Metropolitan Institute of Gerontology, Tokyo, Japan

## Abstract

This 3-year prospective study was conducted to explore whether frequency of participating in the region activity exert independent effect on preventing functional decline among urban Japanese older adults after controlling for potential confounders. We examined a prospective cohort of 2,524 community-dwelling persons, aged 65 years or older, who responded to the baseline mail survey in Toshima ward, Tokyo, Japan in 2014. They were followed for the subsequent 3 years in terms of functional status. Multiple logistic regression models were used to analyze independent effects of frequency of participating in the region activity, such as 1) no participation, 2) no participation in the past year, 3) less than one day per month, 4) few days per month, 5) over one day per week, on functional status, controlling for potential confounders such as age, gender, self-rated health, chronic conditions and social capital at baseline. At baseline, the mean age of 1,261 participants who completely responded to follow-up survey in 2018 was72.1 years (SD=5.0), and 56.9% were women. As results of analyzing, only “over one day per week” was significant predictors of preventing subsequent functional decline even after adjustment for confounders (odds ratios .361; 95% CI .180–.725). Frequency of participating in the region activity over one day per week have effect on preventing functional decline among urban Japanese older adults after controlling for potential confounders.

